# Patients and staff perspectives on hospital ward strain and its impacts on patient care and the workforce: a mixed-methods systematic review

**DOI:** 10.1093/intqhc/mzag101

**Published:** 2026-07-22

**Authors:** Georgia Bercades, Cecilia Vindrola-Padros, David Brealey, Isla Kuhn, Seo Yeon Yoon, Grainne Brady, John Welch, Katharina Kohler

**Affiliations:** Department of Targeted Intervention, University College London, United Kingdom; Department of Targeted Intervention, University College London, United Kingdom; Critical Care Unit, University College London Hospitals NHS Foundation Trust, London, United Kingdom; Cambridge University Medical Library, Cambridge, United Kingdom; Institute of Health Informatics, University College London, United Kingdom; Department of Targeted Intervention, University College London, United Kingdom; Critical Care Unit, University College London Hospitals NHS Foundation Trust, London, United Kingdom; Department of Anaesthesia, Cambridge University Hospital NHS Foundation Trust, Cambridge, United Kingdom

**Keywords:** Hospital ward strain, missed care, patient safety, resilience, staff well-being

## Abstract

**Background:**

Hospital ward strain occurs when care demands exceed available staffing, beds, and resources, posing a significant threat to safe inpatient care. While emergency department and intensive care settings have been extensively studied, ward-level pressures have received less attention, particularly from the perspectives of patients and frontline staff. This review aimed to synthesize current evidence on how hospital ward strain is experienced by staff and patients, and its implications for care quality, safety, and workforce sustainability.

**Methods:**

A mixed methods systematic review was conducted on patient and staff perspectives on hospital ward strain. MEDLINE, CINAHL, PsycINFO, and HMIC were searched for empirical studies published from January 2000 to March 2025. Three reviewers screened titles and abstracts in pairs; full-text assessment was conducted by one reviewer with independent verification of a random 20% sample. The Mixed-Methods Appraisal Tool was used to assess methodological quality. Findings were analysed using Donabedian’s Structure-Process-Outcome (SPO) model and Hollnagel’s Resilience framework.

**Results:**

Of 9,943 studies identified, 16 observational and non-randomized studies met the inclusion criteria, representing diverse healthcare settings across multiple countries. Most studies (87.5%) focused exclusively on staff perspectives; only two included patient experiences. Structural pressures (inadequate staffing, bed capacity constraints) consistently led to process failures (care delays, missed care, coordination breakdowns), resulting in burnout, turnover, quality and safety trade-offs, and compromised patient safety. Ward resilience was mainly reactive: respond and monitor were evident, but anticipate and learn, were mostly absent. Patient acuity was inconsistently reported, limiting cross-study comparison.

**Conclusion:**

Ward strain is a systemic patient safety issue requiring structural solutions beyond staff resilience. Patient perspectives remain underrepresented and must be prioritized in future research. Policymakers should invest in anticipatory and learning resilience capacities through workforce planning, real-time monitoring, and formal learning mechanisms, to ensure sustainable, safe ward care.

## Introduction

Hospital capacity strain reflects the mismatch between available resources and institutional demand, commonly measured by bed occupancy rates or staffing levels [1]. It poses a significant challenge for modern healthcare systems, especially in high-acuity settings like emergency departments (EDs) and intensive care units (ICUs), where it leads to treatment delays, adverse outcomes, and increased mortality [2–9]. However, differences in staffing models, skill-mix, and monitoring structures in general wards limit their transferability, making ward-level strain a comparatively understudied phenomenon.

Ward strain occurs when demand for care, clinical monitoring, bed availability, or equipment exceeds a ward’s operational capacity. Unlike ICUs and EDs, which have continuous electronic monitoring, general wards rely on intermittent manual observations, making the effects of strain harder to capture and more likely to manifest as missed observations, delayed escalation, or reduced care coordination. These effects may significantly impact the quality and safety of patients’ care [10]. Studies show that inpatients with hospital-acquired sepsis experience treatment delays up 3.6 hours as ward census rises [11], and missed vital sign monitoring is common in over 17% of overstretched wards [12]. Higher registered nurse (RN) staffing levels are significantly associated with lower failure-to-respond rates among patients with the most abnormal vital signs [13]. Increased RN staffing is significantly associated with lower patient mortality [14, 15].

Strain has been shown to impact not only patient outcomes but also workforce well-being, contributing to burnout and turnover [16–19]. The COVID-19 pandemic intensified these pressures globally, exposing the vulnerability of ward strain structures [20–22].

Despite the clinical significance, ward strain is largely studied as an operational problem. Research has focused on quantitative metrics such as bed occupancy rates or nurse-to-patient ratios rather than the lived experiences of the people providing or receiving care under strained conditions [23–25]. While valuable for capacity planning, this perspective fails to capture the human dimension of strain and may overlook how it affects staff sustainability, care quality, and safety as perceived by those most directly affected. Patient perspectives, in particular, are largely absent from strain research.

To address these limitations, this review uses two complementary theoretical frameworks. Donabedian’s Structure-Process-Outcome (SPO) model [26] provides a systematic lens for understanding the quality of care as a chain of linked domains: structural inputs (staffing levels, bed occupancy, skill-mix) shape the care process (clinical assessment, communication, coordination, and adaptive behaviors), which in turn affect outcomes (patient safety and quality, workforce well-being). Consistent with Brown *et al.*’s epistemological framework for patient safety research [27], we applied SPO as an interpretative lens to map how structural pressures cascade through care processes to patient and workforce outcomes. Hollnagel’s Resilience Framework [28] examines adaptation across four capacities: respond, monitor, learn, and anticipate, highlighting resilience beyond surface-level workarounds.

This mixed-methods systematic review synthesizes evidence on ward strain from the perspectives of patients and staff, examining the experiences of strain and its implications for care quality, safety, and workforce sustainability. Specifically, we aimed to: (1) identify and characterize studies that examine ward strain from patient and staff perspectives; (2) analyze how structural pressures translate into process changes and outcomes using Donabedian’s model; and (3) examine adaptation mechanisms and resilience capacities using Hollnagel’s Resilience framework.

## Methods

We reported this mixed-methods systematic review according to the Preferred Reporting Items for Systematic Reviews and Meta-Analyses (PRISMA) 2000 statement [29] and registered prospectively with PROSPERO (CRD420251009893). The protocol was developed a priori and is publicly accessible via PROSPERO. The PRISMA 2020 checklist in Supplementary Material 1.

### Search strategy

Four electronic databases, CINAHL, HMIC, MEDLINE, and PsycINFO, were searched using terms related to hospital wards, ward strain, and patient and staff experiences, developed with a medical librarian (IK) at the University of Cambridge. Subject headings were selected individually for each database to maximize coverage. Final searches were completed on 13 March 2025. Full search terms appear in Supplementary Material 2.

### Study selection

Included studies were peer-reviewed, focused on adult ward patients and ward-based staff (nursing, medical, allied health, and non-clinical support staff), and addressed hospital ward strain as defined in the introduction. They reported on patient outcomes (missed care, adverse events, satisfaction) or staff outcomes (job satisfaction and well-being). We included studies that considered ward strain either as a contextual factor or exposure, and prioritized experiences over intervention assessment. Observational, non-randomized, qualitative, and mixed-methods designs were included. Studies focused on ED or ICU settings or non-research publications were excluded. Studies pre-2000 were excluded as they reflected task-based or functional nursing models, which differ structurally from modern team and primary nursing models in divisions of labor, supervision, and accountability [30, 31].

### Data extraction

Search results were combined and duplicates removed using reference management software. Three reviewers (GB1, KK, and SY) screened titles and abstracts in pairs using Rayyan, with each record assessed by two reviewers and discrepancies resolved through discussion. Full-text articles were assessed using a standardized form (Supplementary Material 3). One reviewer (GB1) assessed all full-text articles; a second (GB2) independently verified a random 20% sample. Disagreements were resolved by a third reviewer (KK). Sixteen articles met the criteria.

### Quality assessment of selected articles

Meta-analysis was not feasible given the heterogeneity in study designs. We used the Mixed Methods Appraisal Tool (MMAT) [32] to assess the quality of quantitative, qualitative, and mixed-methods studies, scoring each study on five criteria, as ‘Yes’, ‘No’, or ‘Can’t Tell’ (Supplementary Material 4). Studies meeting all five MMAT criteria were rated high quality; those with four were moderate, and three or fewer were low quality. One reviewer (GB1) assessed the studies, and another (KK) verified the ratings. Low-quality studies were interpreted with caution during synthesis.

### Data synthesis

We structured the data synthesis with Donabedian’s SPO model, selected for consistency and comparability with a previous systematic review [33] and Hollnagel’s Resilience framework to interpret adaptive behaviors and quality and safety trade-offs. Most studies lacked systematic adjustment for patient acuity and complexity, limiting our ability to account for these factors in the synthesis and potentially affecting associations between structural factors and outcomes. Each finding was classified according to both frameworks.

Donabedian’s SPO Framework:

- Structure (resources and set-up):
  - Staffing adequacy and composition
  - Workforce qualifications and skill-mix
  - Bed capacity and physical environments
  - Management support and recognition
  - Workforce sustainability
- Process (actions taken during care):
  - Assessment and response timeliness
  - Direct patient care activities
  - Medication management processes
  - Patient flow and throughput
  - Workload and distribution
- Outcomes (results of care) are organized into three groups:
  - Patient outcomes
    - Medication safety
    - Healthcare-associated complications
    - Care quality perceptions
  - Staff outcomes
    - Mental health and burnout
    - Workforce retention and turnover
    - Staff engagement and support
    - Adaptive behaviors
  - System outcomes
    - Operational efficiency
    - Quality and safety trade-offs

Hollnagel’s Resilience Framework

- Respond: Knowing what to do or respond when something happens.
- Monitor: Knowing what to look for or monitor what is happening.
- Learn: Knowing what has happened or learning from experience.
- Anticipate: Knowing what to expect or anticipating what may happen.

Safety is one of the six Institute of Medicine (IOM) quality domains (alongside effectiveness, patient-centredness, timeliness, efficiency, and equity) [34]. Under conditions of ward strain, tradeoffs between domains are common. For example, staff may prioritize timely task completion (efficiency) at the cost of thorough assessment (safety) while reducing patient-facing communication (patient-centredness).

Two coding boundaries need clarification: operational efficiency was coded as an outcome, but coordination measures represent processes; quality and safety trade-offs were initially coded as processes, but their consequences (missed care, delayed treatment) are better classified as outcomes.

To demonstrate the dual framework coding approach: resident doctors and ward managers working overtime were coded as Structure (staffing), Process (working overtime), and Outcome (burnout risk; short-term efficiency). This reflects the activation of Hollnagel’s respond capacity (working overtime) but not anticipate or learn, showing that immediate solutions can mask structural vulnerability.

Each finding was classified at the micro (individual), meso (department), or macro (system) level and by impact polarity (positive, negative, neutral). One reviewer (GB1) coded the findings, with additional input and review through discussion with a second reviewer (KK). We abridged quotes for clarity, assessed the impact of excluding low-quality studies through sensitivity analyses, and evaluated publication bias by reviewing study characteristics and outcome reporting.

## Results

**Figure 1:**
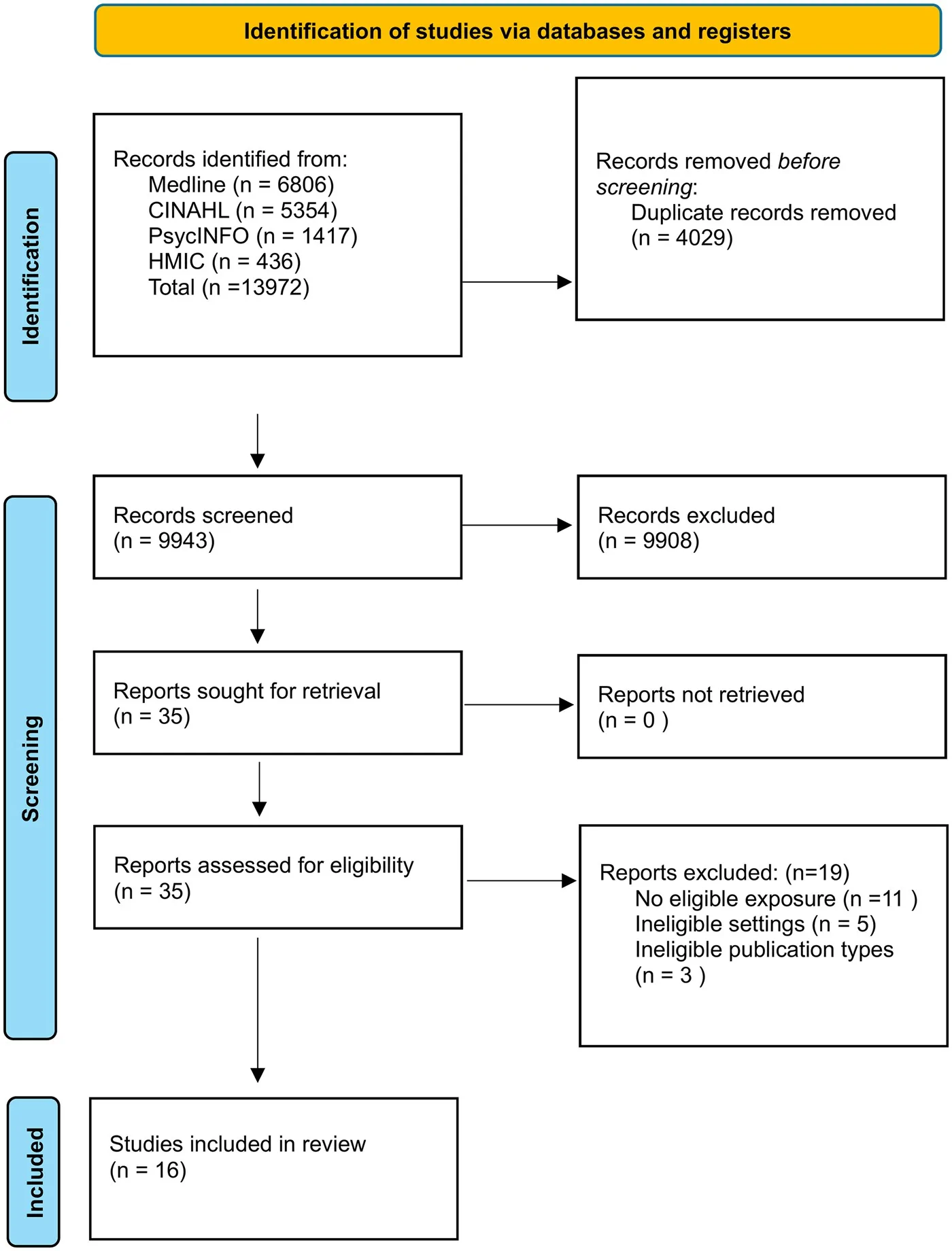
PRISMA 2020 flow diagram for identification, screening, and inclusion of studies in this systematic review.

The initial search yielded 9,943 articles after duplicates were removed. Following the title and abstract screening, 35 articles underwent full-text review for eligibility. Sixteen studies met the inclusion criteria and were included in the final synthesis. The PRISMA flow diagram shows the selection process (Fig. 1).

### Study characteristics

The included studies were published between 2007 and 2024, including six cross-sectional, five interview-based studies, two descriptive correlational, one longitudinal, one mixed-methods, and one ethnographic design. Most of these studies were conducted in high-income countries (HICs), specifically three each from the UK and USA, with additional studies from Australia, Brazil, Canada, Denmark, Germany, Italy, Kuwait, Norway, and South Korea. They took place in various acute care, university, and public hospitals in urban and regional areas. Notably, few studies discussed potential strategies for addressing ward strain; no study implemented or evaluated an intervention. One study was conducted after the first COVID-19 wave; however, it did not explicitly focus on COVID-19, even though the pandemic context may have influenced findings.

Of the studies reviewed, seven focused on nurses, five included clinical and non-clinical staff, two centred on doctors, and two on patients. Medical leaders and hospital executives prioritized operational and financial matters during periods of ward strain [35]. Staff created informal workarounds to maintain workflows, while patients expressed safety concerns and varying levels of satisfaction with their care [36, 37].

Across studies, ward strain was consistently driven by staffing shortages and rising care demands. Staff predominantly reflected reactive capacities outlined in Hollnagel’s Resilience framework, emphasizing immediate action and tracking of evolving conditions, with little evidence of anticipatory or learning strategies [38, 39]. Although organizational support through leadership and peer network support provided temporary buffering, it relied heavily on staff endurance [40, 41].

Most studies were rated moderate to high quality. Sensitivity analysis showed that excluding two lower-quality studies [35, 42] did not significantly alter the main findings, with the proportion of studies reporting negative effects remaining high, regardless of quality (87.5% vs 75%). This indicates robust findings across quality levels. Study characteristics are presented in Table 1.

**Table 1:** Study characteristics of included studies (*n* = 16).

Author, year	Country	Setting	Study design	Population (*n*)	Data collection	Perception of ward strain sign	Impact direction	Resilience potentials	MMAT rating
Al-Kandari, 2008	Kuwait	Regional hospitals (medical & surgical wards)	Cross-sectional	Nurses (780)	Questionnaire	Workforce shortages; High workload	Negative	Monitor–Respond	Moderate
Arogyaswamy, 2022	USA	Academic centres (general wards)	Qualitative	Doctors, nurses, non-clinicians (29)	Interview	Workforce shortages; Delayed or missed care	Negative	Anticipate–Monitor–Respond–Learn	High
Brophy, 2024	Canada	Urban and regional hospitals (general wards)	Qualitative	Hospital support staff (26)	Interview	Workforce shortages; High workload; Burnout; Intention to leave	Negative	Monitor	High
Buerhaus, 2007	USA	Urban and regional hospitals (general wards)	Cross-sectional	Doctors, nurses, non-clinicians (1421)	Questionnaire	Workforce shortages; Delayed or missed care	Negative	Monitor	Low
Catania, 2024	Italy	Public hospitals (surgical wards)	Descriptive & regression	Nurses (1046)	Questionnaire	Workforce shortages; High workload; High turnover	Negative	Monitor–Respond	High
Cho, 2021	South Korea	Urban & regional hospitals (medical & surgical wards)	Cross-sectional secondary analysis	Nurses (10,457)	Questionnaire	Workforce shortages; Delayed or missed care; Trade-offs	Mixed	Monitor–Respond	Moderate
Duffield, 2020	Australia	Public hospitals (general wards)	Mixed methods	Nurses (17)	Interview	Workforce shortages; High workload	Negative	Anticipate–Respond–Learn	High
Goulding, 2015	UK	Acute care hospitals (general wards)	Qualitative	Patients (19)	Interview	Delayed or missed care	Negative	Monitor	High
Happell, 2013	Australia	Acute care hospitals (medical, surgical, paediatric wards)	Qualitative	Nurses (38)	Interview	Workforce shortages; Delayed or missed care	Negative	Monitor–Respond–Learn	High
Krämer, 2016	Germany	Acute care hospitals (general wards)	Longitudinal follow-up	Doctors (95)	Questionnaire	Workforce shortages; High workload	Negative	Monitor	Low
Magalhaes, 2017	Brazil	University hospitals (surgical & clinical units)	Cross-sectional	Nurses and technicians (502); Patients (157 481)	Questionnaire	Workforce shortages; High workload; Adverse events	Negative	Monitor–Respond	High
Mainz, 2024	Denmark	Public university hospitals (medical & surgical wards)	Cross-sectional	Nurses (529)	Questionnaire	Workforce shortages; High workload; Adverse events	Negative	Monitor–Respond	Moderate
Nilsen, 2019	Norway	Hospital trusts (medical & surgical wards)	Qualitative	Doctors and nurses (19)	Interview	Workforce shortages; Delayed or missed care	Negative	Respond–Learn	High
Park, 2018	USA	Acute care hospitals (medical & surgical wards)	Descriptive correlational	Nurses (31 650)	Questionnaire	Workforce shortages; Delayed or missed care	Negative	Monitor–Respond	High
Sanford, 2022	UK	Acute care hospitals (medical & surgical wards)	Qualitative	Doctors & nurses (36)	Ethnography	Workforce shortages; Delayed or missed care; Trade-offs	Mixed	Respond–Learn	High
Teoh, 2021	UK	Acute care hospitals (medical & surgical wards)	Descriptive correlational	Doctors (13 239)	Questionnaire	Workforce shortages; High workload	Negative	Respond–Learn	Moderate

## Framework synthesis

### Structure

We identified 19 distinct evidence codes across the studies and organized them according to structure, process, and outcomes (Table 2). Staffing levels and composition were the predominant structural sources of ward strain [35–50]. Nurse-to-patient ratios ranged from 1:5 to 1:11 [36, 44, 47, 50]. Differences in patient acuity and case mix likely explain the variation, but these factors were inconsistently reported and could not be formally controlled for in our synthesis. Workforce skill-mix deficits included heavy reliance on nursing assistants rather than qualified nurses [36, 43, 50]. One study highlighted the main problem: “Staffing shortages persist as people continue to arrive…regardless of workload…causing significant stress” [48].

**Table 2:** Donabedian structure–process–outcome domain.

Domain	Code	Sub-code	Level	Polarity	Representative quote (abridged)	Study
Structure	Human Resource Infrastructure	Staffing levels & composition	Meso	Negative	Workforce shortage; nurse–patient ratio ranged from 1:4.8 to 1:11	[1–7 ,9–16]
		Workforce qualifications & skill-mix	Macro	Mixed	Between 25.1% and 69% of nurses held BSN degrees	[1, 3, 11, 12]
	Physical Infrastructure	Bed capacity & physical environment	Meso	Negative	Bed occupancy ∼85% (26–30 beds); equipment shortages delayed discharge	[1–3, 5, 8, 15]
	Organizational Structure & Governance	Management support & recognition	Meso	Negative	Staff reported feeling unsupported and lacking recognition	[3, 5, 9, 14]
		Workforce sustainability	Macro	Negative	Underfunding affected long-term workforce stability	[1, 3]
Process	Clinical Assessment & Escalation	Assessment & response timeliness	Micro	Negative	Delayed assessment linked to pressure ulcers, falls, and UTIs	[1, 3–6, 8, 11, 12, 14]
	Care Delivery Process	Direct patient care activities	Micro	Negative	Comfort, emotional support, ambulation, and oral care most frequently missed	[3, 6, 8, 12, 14]
		Medication management processes	Micro	Negative	43% of planned medications delayed by more than 30 min	[1, 9, 10, 14, 15]
	Care Coordination & Flow	Patient flow & throughput	Meso	Negative	Rapid cycling of admissions & discharges	[2, 3, 9, 12, 13]
		Workload & distribution	Meso	Negative	Non-clinical tasks added to nursing responsibilities thus increasing workload	[1–4, 7, 8, 14]
Outcome	System performance	Operational efficiency	Macro	Negative	Increased costs due to reliance on agency staff	[2, 4, 7, 11, 13]
		Quality and safety trade-offs	Meso	Mixed	Staff extended working hours to complete required tasks	[6, 15]
	Staff well-being & sustainability	Mental health & burnout	Micro	Negative	36% of nurses reported job dissatisfaction	[2, 3, 5, 6, 15, 16]
		Workforce retention & turnover	Meso	Negative	Around 30% of nurses intended to leave their positions	[2–4, 6, 9, 11, 14]
	Resilience & adaptive capacity	Staff engagement & support	Micro	Mixed	Engagement associated with job control and management support	[6, 15]
		Adaptive behaviours	Micro	Mixed	Presenteeism used to cover shifts during staff shortages	[6, 15]
	Patient safety outcomes	Healthcare-associated complications	Micro	Negative	Delayed assessment increased risk of ulcers, falls, and UTIs	[1, 11, 14]
		Medication safety	Micro	Negative	Medication labelling skipped to save time during staff shortages	[1, 4, 8, 10, 15]
	Patient experience & satisfaction	Care quality perception	Micro	Negative	Increased workload negatively affected patient care quality	[3, 5, 7–11, 13, 14, 16]

### Process

The most immediate effect of excessive demand is increased workload, leading to process-level failures, including delayed assessment [35–37, 41, 43, 44, 47, 50], missed care [37, 41, 43, 45, 47], compromised coordination [38, 43, 45, 48, 49], and inequitable workload distribution [35, 37–39, 41, 43, 50].

### Outcomes

Patient outcomes—These failures resulted in delays or omissions in medication delivery [41, 42, 46, 48, 50], less patient interaction [41, 45, 47], and reduced care delivery [36, 37, 39–44, 49]. Delayed assessment increased the incidence of adverse events, including pressure ulcers, urinary tract infections, and falls [41, 45, 50]. Patients and families expressed frustration with delays, communication gaps, and perceived staff inattention [50]. System outcomes—Bed occupancy exceeding 85% and equipment shortages further delayed admissions and discharges [37, 38, 43, 44, 46]. Staff outcomes—These structural and process-level inefficiencies contributed to negative outcomes, including burnout and turnover [36, 38, 40, 41, 43, 44, 46–48].

### Adaptive behaviors and resilience capacities

**Figure 2:**
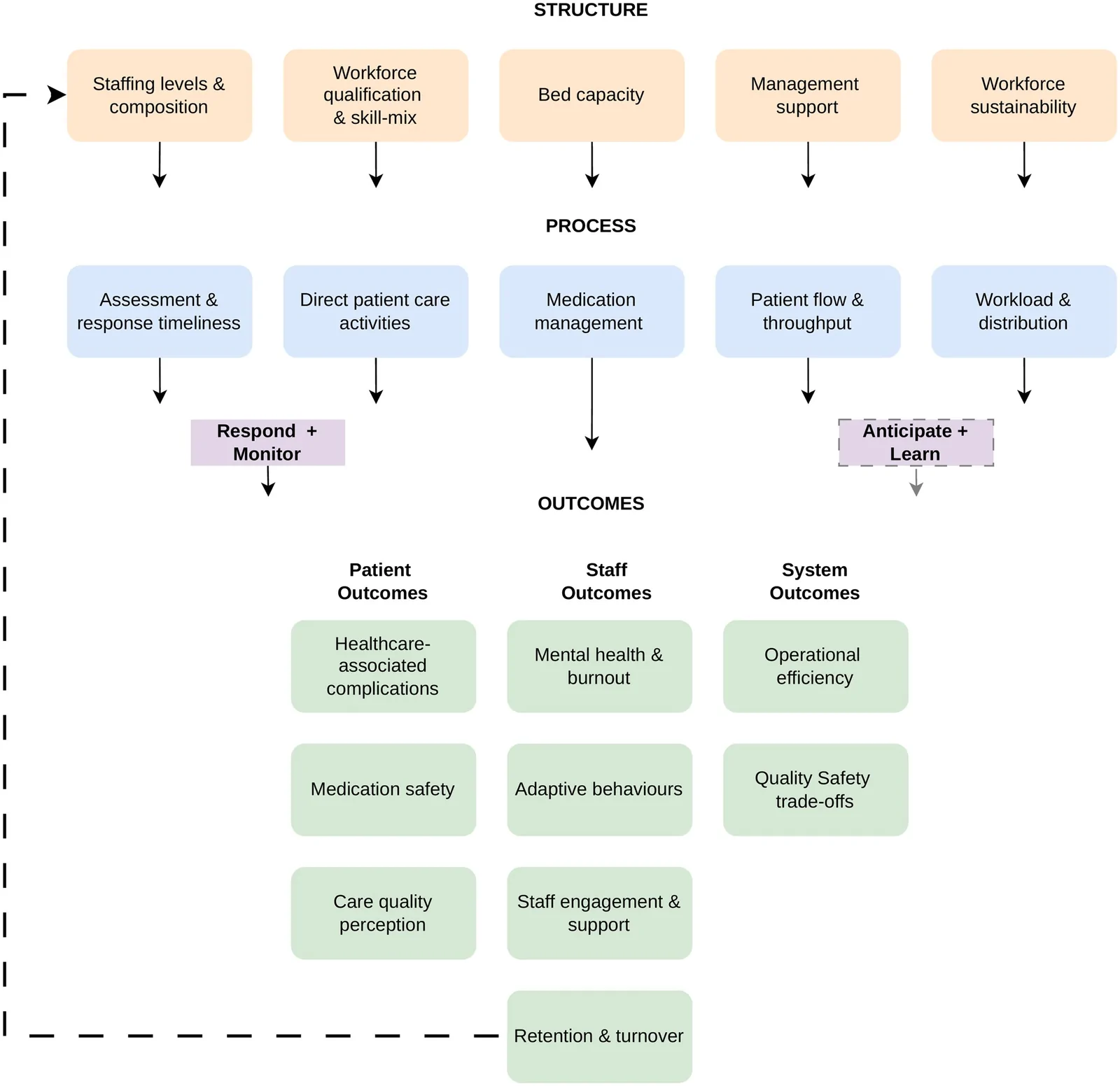
Conceptual Model of Hospital Ward Strain integrating Donabedian’s Structure–Process–Outcome model and Hollnagel’s Resilience framework. The figure shows the SPO cascade from structural deficits through process failures to patient, staff, and systems outcomes; Hollnagel’s four capacities mapped onto the process layer as two paired boxes (Respond + Monitor and Anticipate + Learn), and the self-reinforcing feedback loop from turnover back to staffing shortages. The dashed outline of the Anticipate + Learn box reflects this review’s finding that anticipatory and learning resilience capacities were largely underdeveloped in current ward practice, in contrast to the more consistently applied Respond + Monitor capacities.

Adaptive behaviors and quality and safety trade-offs, instances where staff prioritized one IOM quality domain at the cost of another, showed mixed effects. Staff adaptations such as presenteeism, skipped breaks, cross-covering roles, overtime, and reliance on agency staff sustained ward function in the short term but masked underlying fragility [38, 46, 47]. These actions led to ongoing fatigue and higher staff turnover, worsening the very weaknesses they sought to compensate for. Although management support occasionally buffered the impact of strain, it was insufficient to address core structural deficits (Fig. 2).

While most approaches were reactive, a few studies found proactive strategies that use Hollnagel’s anticipate and learn capacities, such as predictive analytics using inflow and outflow data, nurse leaders’ expertise in bed management, and real-time monitoring dashboards [38, 39, 48].

## Discussion

### Statement of principal findings

Across 16 studies, staffing shortages and high bed occupancy triggered a cascade of process failures, including missed care, delayed escalation, and disrupted patient flow. These results highlight the interdependence of SPO within Donabedian’s model, aligned with wider evidence on patient and quality safety and healthcare system resilience [28].

Hollnagel’s Resilience framework found that wards mainly use reactive strategies, with limited proactive anticipation or learning. This imbalance makes hospitals prone to recurring crises, as healthcare organizations often focus on urgent needs instead of long-term learning or anticipatory planning [51]. Without investment in proactive strategies, reactive adaptation becomes the structural norm, a position that is costly, fragile, and harmful to staff and patients.

### Strengths and limitations

To our knowledge, this review is the first to synthesize evidence on ward strain using Donabedian’s model and Hollnagel’s Resilience framework. This dual-framework approach moves beyond descriptive accounts of staffing pressures to examine structural deficits that cascade through care processes and how resilience capacities shape outcomes across professional groups. The use of a prospectively registered protocol, PRISMA 2020 reporting, and sensitivity analysis further strengthens the methodological rigour of this review.

Most included studies were from HIC settings with team nursing models, limiting generalizability. Resilience capacities may be incompletely described if not detailed in source reports. Only published literature was included, increasing publication bias risk. Design heterogeneity prevented meta-analysis, and cross-sectional designs limit causal inference. Patient acuity was inconsistently reported, limiting its role as a potential confounder. The SPO model was used interpretively, as domain boundaries overlapped, quality and safety trade-offs affected both process and outcome, and causal links were not empirically confirmed.

### Interpretation within the context of the wider literature

Our findings contribute to the existing research on hospital capacity strain by highlighting ward-level experiences, which have been less represented than ED and ICU research. Previous systematic reviews [33, 52] have documented similar patterns of delayed care and adverse outcomes in high-pressure environments, suggesting that these patterns are consistent across ward-level staffing metrics, workload indicators, burnout, and job satisfaction. Studies show that staffing shortages and high bed occupancy create a cycle where worsening conditions lead to more turnover and less capacity [53, 54]. Implementing proactive capacity planning, such as flexible staffing and real-time monitoring of beds and workload, could help mitigate the negative effects of ward strain [55].

Only two studies included patient perspectives: one offering qualitative insights and the other analyzing over 150,000 survey responses. This shows the gap noted in the introduction. Patients can identify risks unnoticed by staff or conventional metrics [56, 57], yet their perspectives remain marginalized in ward strain research. Embedding patient voices could strengthen anticipatory capacity within Hollnagel’s framework, shifting wards from reactive adaptation toward proactive learning [58]. However, evidence remains sparse and heterogeneous. Future studies should prioritize participatory methods such as co-design [59], and digital reporting [60] to integrate patients’ perspectives into ward safety protocols.

Ward strain affects all staff but is experienced differently by role. Executives prioritize efficiency [35], clinicians focus on quality and safety [40, 46], while support staff perform essential but often unrecognized tasks [43]. Each role faces distinct pressures that reflect its hierarchical position and create risks of misaligned priorities and communication gaps [61]. Interdisciplinary forums have been proposed to address these issues, but their effectiveness under strain needs further investigation [62].

Ward strain varies across different contexts, regions, and organizational cultures. HICs generally have more resources and team nursing models that support quick adaptation [63]. Low- and middle-income countries (LMICs) often face longer periods of resource limitations and higher nurse-to-patient ratios, with less flexibility and fewer escalation pathways [64]. Hierarchical cultures can inhibit frontline staff from escalating concerns, delaying intervention, while collaborative cultures promote open communication and more effective adaptation [65, 66]. Limited data restrict global applicability, highlighting the need for comparative research on governance, resource allocation, and cultural norms.

### Implications for policy, practice, and research

Workforce planning and bed management should consider safety metrics like staffing ratios, bed occupancy, and missed care indicators, along with operational and financial factors [67, 68]. Real-time metrics should be used to trigger prompt interventions, and investment in predictive workload monitoring and surge planning would support proactive resilience capacities largely absent from current ward practice [67]. Where not already established, formal learning mechanisms, such as after-action reviews, should be institutionalized; where they exist, consistent application and systemic use of their outputs should be prioritized [69].

Organizations must move beyond dependence on staff improvisation as a safety mechanism [70, 71]. Leadership, teamwork, and structural safeguards are essential to support staff well-being and minimize quality and safety trade-offs [72]. Protocols for medication administration and patient assessment must be consistently followed under conditions of strain, as delays were reported process failures in this review. Accessible mental health support must be available to staff experiencing cumulative effects of sustained strain [73].

Building on the reactive resilience pattern identified in this review, future studies should prioritize measuring anticipatory and learning resilience capacities over responsive adaptations and workarounds [28, 38, 39]. Comparative research should include cross-sectional studies on ward strain across resource levels, with qualitative methods exploring cultural attitudes toward escalation and adaptation in healthcare settings.

## Conclusions

Ward strain is a result of systemic failure, not an inevitability, but a tolerated condition. Patient warnings of risk remain underused, leading to preventable harm [57]. Hospitals must move beyond early-warning systems alone, embedding structural and procedural safeguards that enable proactive rather than reactive responses [74]. Resilience must be a strategic priority, as patients and staff deserve systems that protect them rather than relying on their endurance.

## Supplementary Material

mzag101_Supplementary_Data

## Data Availability

The data underlying this article are available in the article and in its online supplementary material.
