# Supplementary material for: Patients and staff perspectives on hospital ward strain and its impacts on patient care and the workforce: a mixed-methods systematic review
Source: Int J Qual Health Care. 2026 Jul 22;38(3):mzag101. doi: 10.1093/intqhc/mzag101 (PMC13455353; doi:10.1093/intqhc/mzag101)
Supplement: Supplementary file 1 [file mzag101_supplementary_data.zip › Supplementary_Material_File_4.docx]

**Supplementary Material File 4. Mixed Methods Appraisal Tool (MMAT)**

**Table 4. Mixed Methods Appraisal Tool (MMAT)**

| **Author, Year, Study ID** | **Citation** | **Screening Questions (All)** | **Study Design** | **QUAL (5)** | **RCT (5)** | **Non-Randomised Studies (5)** | **Quantitative Descriptive Studies (5)** | **Mixed Methods (5)** | **Total Score** |
| --- | --- | --- | --- | --- | --- | --- | --- | --- | --- |
| Al-Kandari, 2008 (1) | Al-Kandari F, Thomas D. Perceived adverse patient outcomes correlated to nurses' workload in medical and surgical wards of selected hospitals in Kuwait. J Clin Nurs. 2009;18(4):581-90. | Y | Quantitative Descriptive | N/A | N/A | N/A | Y/Y/Y/Y/Can't tell | N/A | 4 |
| Arogyaswamy, 2022 (2) | Arogyaswamy S, Vukovic N, Keniston A, Apgar S, Bowden K, Kantor MA, et al. The Impact of Hospital Capacity Strain: a Qualitative Analysis of Experience and Solutions at 13 Academic Medical Centers. J Gen Intern Med. 2022;37(6):1463-74. | Y | Qualitative | Y/Y/Y/Y/Y | N/A | N/A | N/A | N/A | 5 |
| Brophy, 2024 (3) | Brophy JT, Keith MM, Hurley M, Slatin C. Running on Empty: Ontario Hospital Workers' Mental Health and Well-Being Deteriorating Under Austerity-Driven System. New Solut. 2024;34(3):182-97. | Y | Qualitative | Y/Y/Y/Y/Y | N/A | N/A | N/A | N/A | 5 |
| Buerhaus, 2007 (4) | Buerhaus PI, Donelan K, Ulrich BT, Norman L, DesRoches C, Dittus R. Impact Of The Nurse Shortage On Hospital Patient Care: Comparative Perspectives, Physicians and hospital executives often do not associate nurses with patient safety and early detection of complications. Health Affairs. 2007;26(3):853-62. | Y | Quantitative Descriptive | N/A | N/A | N/A | Y/Can't tell/Yes/Can't tell/Yes | N/A | 3 |
| Catania, 2024 (5) | Catania G, Zanini M, Cremona MA, Landa P, Musio ME, Watson R, et al. Nurses' intention to leave, nurse workload and in-hospital patient mortality in Italy: A descriptive and regression study. Health Policy. 2024;143:105032. | Y | Quantitative Descriptive | N/A | N/A | N/A | Y/Y/Y/Y/Y | N/A | 5 |
| Cho, 2021 (6) | Cho H, Han K, Ryu E, Choi E. Work Schedule Characteristics, Missed Nursing Care, and Organizational Commitment Among Hospital Nurses in Korea. J Nurs Scholarsh. 2021;53(1):106-14. | Y | Quantitative Descriptive | N/A | N/A | N/A | Y/Can't tell/Y/Y/Y | N/A | 4 |
| Duffield, 2020 (7) | Duffield C, Roche MA, Wise S, Debono D. Harnessing ward-level administrative data and expert knowledge to improve staffing decisions: A multi-method case study. J Adv Nurs. 2020;76(1):287-96. | Y | Mixed-methods | N/A | N/A | N/A | N/A | Y/Y/Y/Y/Y | 5 |
| Goulding, 2013 (8) | Goulding L, Adamson J, Watt I, Wright J. Lost in hospital: a qualitative interview study that explores the perceptions of NHS inpatients who spent time on clinically inappropriate hospital wards. Health Expect. 2015;18(5):982-94. | Y | Qualitative | Y/Y/Y/Y/Y | N/A | N/A | N/A | N/A | 5 |
| Happell, 2013 (9) | Happell B, Dwyer T, Reid-Searl K, Burke KJ, Caperchione CM, Gaskin CJ. Nurses and stress: recognizing causes and seeking solutions. J Nurs Manag. 2013;21(4):638-47. | Y | Qualitative | Y/Y/Y/Y/Y | N/A | N/A | N/A | N/A | 5 |
| Krámer, 2016 (10) | Krámer T, Schneider A, Spiess E, Angerer P, Weigl M. Associations between job demands, work-related strain and perceived quality of care: a longitudinal study among hospital physicians. Int J Qual Health Care. 2016;28(6):824-9. | Y | Quantitative Descriptive | N/A | N/A | N/A | Y/Can't tell/Yes/Can't tell/Yes | N/A | 3 |
| Magalhaes, 2017 (11) | Magalhaes AMM, Costa DGD, Riboldi CO, Mergen T, Barbosa ADS, Moura G. Association between workload of the nursing staff and patient safety outcomes. Rev Esc Enferm USP. 2017;51:e03255. | Y | Quantitative Descriptive | N/A | N/A | N/A | Y/Y/Y/Y/Y | N/A | 5 |
| Mainz, 2024 (12) | Mainz H, Tei R, Andersen KV, Lisby M, Gregersen M. Prevalence of missed nursing care and its association with work experience: A cross-sectional survey. Int J Nurs Stud Adv. 2024;6:100196. | Y | Quantitative Descriptive | N/A | N/A | N/A | Y/Can't tell/Y/Y/Y | N/A | 4 |
| Nilsen, 2019 (13) | Nilsen SM, Asheim A, Tøndel G. Health personnel's perceptions of pressure on capacity in the specialist health services. Tidsskr Nor Laegeforen. 2019;139(18). | Y | Qualitative | Y/Y/Y/Y/Y | N/A | N/A | N/A | N/A | 5 |
| Park, 2018 (14) | Park SH, Hanchett M, Ma C. Practice Environment Characteristics Associated With Missed Nursing Care. J Nurs Scholarsh.2018;50(6):722-30. | Y | Quantitative Descriptive | N/A | N/A | N/A | Y/Y/Y/Y/Y | N/A | 5 |
| Sanford, 2022 (15) | Sanford N, Lavelle M, Markiewicz O, Reedy G, Rafferty AM, Darzi A, et al. Capturing challenges and trade-offs in healthcare work using the pressures diagram: An ethnographic study. Appl Ergon. 2022;101:103688. | Y | Qualitative | Y/Y/Y/Y/Y | N/A | N/A | N/A | N/A | 5 |
| Teoh, 2021 (16) | Teoh KR-H, Hassard J, Cox T. Doctors’ working conditions, wellbeing and hospital quality of care: A multilevel analysis. Safety Science. 2021;135. | Y | Quantitative Descriptive | N/A | N/A | N/A | Y/Y/Y/Can't tell/Y | N/A | 4 |
