# Supplementary material for: Patients and staff perspectives on hospital ward strain and its impacts on patient care and the workforce: a mixed-methods systematic review
Source: Int J Qual Health Care. 2026 Jul 22;38(3):mzag101. doi: 10.1093/intqhc/mzag101 (PMC13455353; doi:10.1093/intqhc/mzag101)
Supplement: Supplementary file 1 [file mzag101_supplementary_data.zip › Supplementary_Material_File_3.docx]

**Supplementary Material File 3. Data extraction table**

**Table 3. Data extraction table**

| **First Author, Year** | **Country** | **Study objectives as stated by authors** | **Type of study** | **Setting** | **Study Design** | **Participants** | **Y/N** | **Intervention or Exposure** | **Y/N** | **Outcome measures** | **Y/N** | **Decision** |
| --- | --- | --- | --- | --- | --- | --- | --- | --- | --- | --- | --- | --- |
| Al Kandari, 2009 (1) | Kuwait | 1. To identify the perceived adverse patient outcomes as related to nurses' workload; 2. To assess nurses' perception of variables contributing to the workload and adverse patient outcomes. | QUAN | Regional | Cross-sectional | 780 nurses | Y | Ward strain | Y | Patient outcome - Adverse events or incidents | Y | Include |
| Arogyaswamy, 2022 (2) | USA | 1. To explore interventions to address hospital capacity strain and the perceived impact of these interventions; 2. To understand how hospital capacity strain impacts patients, the workforce, and other institutional priorities. | QUAL | Academic centres | Qualitative-Interviews | 20 physician leaders, 9 non-physicians (nursing, social work, business leaders) = 29 | Y | Ward strain | Y | Staff experience - Job satisfaction | Y | Include |
| Brophy, 2024 (3) | Canada | 1. To gain insights into the perceptions, concerns and experiences of the less highly paid hospital staff in Ontario's under-resourced and inherently hierarchical health care system. | QUAL | Urban and regional | Qualitative-Interviews | 15 registered practical nurses, 3 cleaners, 5 clerks, 1 physiotherapy assistant, 1 porter, 1 personal support worker = 26 | Y | Ward strain | Y | Staff experience - Well being | Y | Include |
| Buerhaus, 2007 (4) | USA | 1. To examine the impact of the nurse shortage on hospital patient care, focusing on perceptions from registered nurses, physicians and hospital executives. 2. To understand how the shortage affects care processes, hospital capacity, nursing practice, and the Institute of Medicine's (IOM) six aims for improving health care systems. | QUAN | Urban and regional | Cross-sectional | 400 physicians, 1697 nurses, 222 chief nursing officers, 142 chief executives | Y | Ward strain | Y | Staff experience - Job satisfaction | Y | Include |
| Catania, 2024 (5) | Italy | 1. To examine the relationship between nurses' intention to leave their positions, nurse workload, and in-hospital patient mortality in Italy. | QUAN | Public | Cross-sectional | 1518 nurses | Y | Ward strain | Y | Staff experience - Job satisfaction | Y | Include |
| Cho, 2021 (6) | South Korea | 1. To describe Korean nurses' work schedule characteristics and identifies their components to investigate associations of work schedule components with missing nursing care and organisational commitment. | QUAN | Urban and regional | Cross-sectional secondary analysis | 1057 nurses | Y | Ward strain | Y | Staff experience - Well being | Y | Include |
| Duffield, 2020 (7) | Australia | 1. To investigate the impact of changes to bed configuration on nurses' workload | MIXED Method | Public | Mixed method | 17 nurses | Y | Ward strain | Y | Staff experience - Job satisfaction | Y | Include |
| Goulding, 2015 (8) | UK | 1. To explore patients' perspectives of the quality and safety of care received during their inpatient stay on a clinically inappropriate hospital ward. | QUAL | Acute care | Qualitative-Interviews | 19 patients | Y | Ward strain | Y | Patient outcome - Patient Satisfaction | Y | Include |
| Happell, 2013 (9) | Australia | 1. To identify nurses' occupational stressors, and ways in which the organisation may be able to help reduce nurse occupation-related stress. | QUAL | Acute care | Qualitative-Interviews | 38 nurses from paediatric, surgical and medical wards | Y | Ward strain | Y | Staff experience - Job satisfaction | Y | Include |
| Kramer, 2016 (10) | Germany | 1. To determine prospective associations between three job demands, work-related strain and perceived quality of care. | QUAN | Acute care | Longitudinal follow-up | 95 physicians | Y | Ward strain | Y | Staff experience - Job satisfaction | Y | Include |
| Mainz, 2024 (11) | Denmark | 1. To investigate the prevalence and reasons for missed nursing care and whether nurses' work experience was associated with missed nursing care. | QUAN | Public university | Cross-sectional | 529 nurses | Y | Ward strain | Y | Patient outcome - Missed care | Y | Include |
| Magalhaes, 2017 (12) | Brazil | 1. To describe the workload of the nursing team and relate it with patient safety outcomes in clinical and surgical inpatient units of a university hospital. | QUAN | University | Cross-sectional | 157,481 patients, 502 nurses | Y | Ward strain | Y | Patient outcome - Patient Satisfaction | Y | Include |
| Nilsen, 2019 (13) | Norway | 1. To examine how healthcare personnel experience and deal with pressure on capacity in the somatic specialist health services. | QUAL | Hospital trusts | Qualitative-Interviews | 19 managers, doctors and nurses | Y | Ward strain | Y | Staff experience - Job satisfaction | Y | Include |
| Park, 2018 (14) | USA | 1. To examine the relationship between the quality of nurse practice environment and missed nursing care; 2. To identify which characteristics of the nurse practice environment are more likely associated with missed nursing care. | QUAN | Acute care | Descriptive correlational | 31650 nurses | Y | Ward strain | Y | Patient outcome - Missed care | Y | Include |
| Sanford, 2022 (15) | UK | 1. To identify and categorise the types of pressures that healthcare workers experience; 2. To understand what risk trade-off decisions are made in response to pressures; 3. To analyse the implications of risk trade-offs for the quality and safety of care. | QUAL | Acute care | Qualitative-Ethnography | 88.5 hours of observations (2 surgical wards, 1 older adult ward, 1 acute assessment unit, 1 critical care unit) | Y | Ward strain | Y | Staff experience - Job satisfaction | Y | Include |
| Teoh, 2021 (16) | UK | 1. To examine the relationship between hospital doctor's working conditions and their well-being and how these factors are associated with hospital-level quality of care outcomes. | QUAN | Acute care | Descriptive correlational | 13,239 doctors | Y | Ward strain | Y | Staff experience - Well being | Y | Include |
