# Supplementary material for: Patients and staff perspectives on hospital ward strain and its impacts on patient care and the workforce: a mixed-methods systematic review
Source: Int J Qual Health Care. 2026 Jul 22;38(3):mzag101. doi: 10.1093/intqhc/mzag101 (PMC13455353; doi:10.1093/intqhc/mzag101)
Supplement: Supplementary file 1 [file mzag101_supplementary_data.zip › Supplementary_Material_File_2.docx]

**Supplementary Material File 2. Search strategies**

| Medline via Ovid | 6806 |
| --- | --- |
| CINAHL via EbscoHost | 5345 |
| PsycINFO via EbscoHost | 1417 |
| HMIC via Ovid | 436 |
| Total | 13972 |
| Total removed via deduplication | 3731 |
| Total after deduplicated | 10061 |

Date limit of 2000 to present

Limited to English language

Searches run 13^th^ March 2025

Medline

Ovid MEDLINE(R) and Epub Ahead of Print, In-Process, In-Data-Review & Other Non-Indexed Citations, Daily and Versions <1946 to March 12, 2025>

<https://ovidsp.ovid.com/athens/ovidweb.cgi?T=JS&NEWS=N&PAGE=main&SHAREDSEARCHID=YicLKfV5Onw38X5XGuFyvWUJRjRK5xYKbtiPbjWYh7VEzr4vr1eXU39dDiTNfXw4>

1	((ward* or hospital* or inpatient* or bed or department*) adj2 (strain or strained or strains or capacity or overcapacity or overcrowding or occupancy or workload or overboarding or busyness or busy or demand* or pressur*)).ti,ab,kw,kf.	10434

2	(outcome* or impact* or effect or affect* or staff or personnel or patient* or care or quality).ti,ab,kw,kf.	16167708

3	1 and 2	8813

4	((eye or bacteria* or disease or heart or muscle or muscular or musculoskeletal or cardiovascular or heart or pulmonary or microbiology* or mrsa or ecoli or candida) and strain*).ti,ab,kw,kf.	333152

5	(editorial or comment or letter).pt.	2316826

6	3 not (4 or 5)	8227

7	limit 6 to english language	7711

8	limit 7 to yr="2000 -Current"	6806

PsycINFO

| **#** | **Query** | **Limiters/Expanders** | **Last Run Via** | **Results** |
| --- | --- | --- | --- | --- |
| S1 | ((ward* or hospital* or inpatient* or bed or department*) n2 (strain or strained or strains or capacity or overcapacity or overcrowding or occupancy or workload or overboarding or busyness or busy or demand* or pressur*)) | Expanders - Apply equivalent subjects Search modes - Proximity | Interface - EBSCOhost Research Databases Search Screen - Basic Search Database - APA PsycInfo | 2,362 |
| S2 | (outcome* or impact* or effect or affect* or staff or personnel or patient* or care or quality) | Expanders - Apply equivalent subjects Search modes - Proximity | Interface - EBSCOhost Research Databases Search Screen - Basic Search Database - APA PsycInfo | 3,421,239 |
| S3 | S1 AND S2 | Expanders - Apply equivalent subjects Search modes - Proximity | Interface - EBSCOhost Research Databases Search Screen - Basic Search Database - APA PsycInfo | 2,243 |
| S4 | ((eye or bacteria* or disease or heart or muscle or muscular or musculoskeletal or cardiovascular or heart or pulmonary or microbiology* or mrsa or ecoli or candida) and strain*) | Expanders - Apply equivalent subjects Search modes - Proximity | Interface - EBSCOhost Research Databases Search Screen - Basic Search Database - APA PsycInfo | 10,161 |
| S5 | s3 not s4 | Expanders - Apply equivalent subjects Search modes - Proximity | Interface - EBSCOhost Research Databases Search Screen - Basic Search Database - APA PsycInfo | 2,219 |
| S6 | s3 not s4 | Limiters - Publication Year: 2000-2025 Expanders - Apply equivalent subjects Search modes - Proximity | Interface - EBSCOhost Research Databases Search Screen - Basic Search Database - APA PsycInfo | 1,498 |
| S7 | s3 not s4 | Limiters - Publication Year: 2000-2025 Expanders - Apply equivalent subjects Narrow by Language: - english Search modes - Proximity | Interface - EBSCOhost Research Databases Search Screen - Basic Search Database - APA PsycInfo | 1,417 |

CINAHL

| **#** | **Query** | **Limiters/Expanders** | **Last Run Via** | **Results** |
| --- | --- | --- | --- | --- |
| S1 | TI((ward* or hospital* or inpatient* or bed or department*) n2 (strain or strained or strains or capacity or overcapacity or overcrowding or occupancy or workload or overboarding or busyness or busy or demand* or pressur*)) or AB((ward* or hospital* or inpatient* or bed or department*) n2 (strain or strained or strains or capacity or overcapacity or overcrowding or occupancy or workload or overboarding or busyness or busy or demand* or pressur*)) | Expanders - Apply equivalent subjects  Search modes - Proximity | Interface - EBSCOhost Research Databases  Search Screen - Advanced Search  Database - CINAHL | 6,816 |
| S2 | TI(outcome* or impact* or effect or affect* or staff or personnel or patient* or care or quality) or AB(outcome* or impact* or effect or affect* or staff or personnel or patient* or care or quality) | Expanders - Apply equivalent subjects  Search modes - Proximity | Interface - EBSCOhost Research Databases  Search Screen - Advanced Search  Database - CINAHL | 3,960,395 |
| S3 | S1 AND S2 | Expanders - Apply equivalent subjects  Search modes - Proximity | Interface - EBSCOhost Research Databases  Search Screen - Advanced Search  Database - CINAHL | 5,861 |
| S4 | TI((eye or bacteria* or disease or heart or muscle or muscular or musculoskeletal or cardiovascular or heart or pulmonary or microbiology* or mrsa or ecoli or candida) and strain*) or AB((eye or bacteria* or disease or heart or muscle or muscular or musculoskeletal or cardiovascular or heart or pulmonary or microbiology* or mrsa or ecoli or candida) and strain*) | Expanders - Apply equivalent subjects  Search modes - Proximity | Interface - EBSCOhost Research Databases  Search Screen - Advanced Search  Database - CINAHL | 21,666 |
| S5 | s3 not s4 | Expanders - Apply equivalent subjects  Search modes - Proximity | Interface - EBSCOhost Research Databases  Search Screen - Advanced Search  Database - CINAHL | 5,701 |
| S6 | s3 not s4 | Limiters - Publication Date: 20000101-20251231  Expanders - Apply equivalent subjects  Search modes - Proximity | Interface - EBSCOhost Research Databases  Search Screen - Advanced Search  Database - CINAHL | 5,345 |
| S7 | s3 not s4 | Limiters - Publication Date: 20000101-20251231  Expanders - Apply equivalent subjects  Narrow by Language: - english  Search modes - Proximity | Interface - EBSCOhost Research Databases  Search Screen - Advanced Search  Database - CINAHL | 5,345 |

HMIC (Health Management Information Consortium)

Ovid MEDLINE(R) and Epub Ahead of Print, In-Process, In-Data-Review & Other Non-Indexed Citations, Daily and Versions <1946 to March 12, 2025>

<https://ovidsp.ovid.com/athens/ovidweb.cgi?T=JS&NEWS=N&PAGE=main&SHAREDSEARCHID=BNMQSMH6snxApueugV0DBIplerna9A7I7lmaNsZQ2GbPlACwpBu36ylab8XDKpJ0>

1	((ward* or hospital* or inpatient* or bed or department*) adj2 (strain or strained or strains or capacity or overcapacity or overcrowding or occupancy or workload or overboarding or busyness or busy or demand* or pressur*)).ti,ab,kw,kf.	10434

2	(outcome* or impact* or effect or affect* or staff or personnel or patient* or care or quality).ti,ab,kw,kf.	16167708

3	1 and 2	8813

4	((eye or bacteria* or disease or heart or muscle or muscular or musculoskeletal or cardiovascular or heart or pulmonary or microbiology* or mrsa or ecoli or candida) and strain*).ti,ab,kw,kf.	333152

5	(editorial or comment or letter).pt.	2316826

6	3 not (4 or 5)	8227

7	limit 6 to english language	7711

8	limit 7 to yr="2000 -Current"
